# The Medical News Visiting List for 1893

**Published:** 1892-12

**Authors:** 


THE MEDICAL NEWS VISITING LIST. 1893.—Thirty patients per week. Philadelphia: Lea Brothers & Co.

WE ARE in receipt of the above little book. It is thumb-lettered and contains 104 pages for the daily visiting record, accommodating thirty names to the page, together with several pages each for clinical record, consultation practice, obstetric engagements, vaccinations, death register, addresses of patients and nurses, and cash account. The first four pages are devoted to notes upon the signs of dentition, how to find the day of confinement, thermometric scales, weights and measures, comparative scales, examination of the urine, important incompatibles, artificial respiration, table of eruptive fevers, poisons and antidotes, table of doses, therapeutic reminders and ligation of arteries. C. E. J.

